# The Role of Endothelial Glycocalyx in the Pathophysiology of Chronic Kidney Disease and Hypertension: From Molecular Mechanisms to Clinical Biomarkers

**DOI:** 10.3390/life16040642

**Published:** 2026-04-10

**Authors:** Ana Marinčić Žagar, Nikolina Kolobarić, Petar Šušnjara, Justina Mihaljević, Zrinka Mihaljević, Ines Drenjančević

**Affiliations:** 1 Department of Physiology and Immunology, Faculty of Medicine Osijek, Josip Juraj Strossmayer University of Osijek, 31000 Osijek, Croatia; amarincic@mefos.hr (A.M.Ž.); nbdujmusic@mefos.hr (N.K.); zmihaljevic@mefos.hr (Z.M.); 2 Department of Interdisciplinary Sciences, Faculty of Kinesiology Osijek, Josip Juraj Strossmayer University of Osijek, 31000 Osijek, Croatia; psusnjara@kifos.hr; 3 Department of Radiology, Clinical Hospital Centre Osijek, 31000 Osijek, Croatia; justinamihaljevic@gmail.com

**Keywords:** biomarkers, endothelium, glycocalyx, hypertension, oxidative stress, renal insufficiency, chronic

## Abstract

Hypertension and chronic kidney disease (CKD) are closely linked conditions and represent common global health problems. Hypertension is a leading risk factor for cardiovascular disease, which is the main cause of mortality in CKD. Endothelial injury underlies the etiopathogenesis of both hypertension and CKD. The endothelial glycocalyx (eGC) is a dynamic, negatively charged, carbohydrate-rich layer that covers the luminal surface of endothelial cells. Its primary physiological function is to protect the endothelium, including the regulation of vascular permeability and homeostasis. Damage to the eGC, known as “shedding”, is an early predictor of endothelial dysfunction and is driven by oxidative stress and low-grade inflammation. In hypertension, loss of eGC integrity—often impaired by a high-salt diet—can reduce the bioavailability of nitric oxide (NO) and increase arterial stiffness. Similarly, in CKD, uremic toxicity, hypertension, and inflammation damage the eGC, resulting in increased permeability, albuminuria, and higher cardiovascular risk. This review summarizes current evidence and underscores the potential of eGC shedding markers, especially syndecan 1 (SDC-1) and hyaluronic acid (HA), as early predictors of vascular risk and disease progression in hypertension and CKD.

## 1. Introduction

The endothelium is an active barrier between the vascular wall and circulating blood, consisting of a monolayer of endothelial cells (ECs) [1]. ECs synthesize various mediators that influence vascular hemodynamics [2] and blood coagulation [3]. The endothelium was long considered an inert barrier, but later studies demonstrate that it plays important active roles, such as regulating vascular tone to maintain the balance of vasoactive molecules, controlling vascular permeability, and regulating inflammatory processes and smooth muscle cell proliferation. It is also involved in hemostasis and angiogenesis [1,4,5]. Therefore, the endothelium is considered a dynamic metabolic organ, and an important factor in maintaining vascular homeostasis [3,6,7]. The endothelial glycocalyx (eGC) was first identified and named through chemical analysis, which revealed its high sugar content [8]. The eGC plays an important role in maintaining vascular permeability and is a main regulator of endothelial function; however, its primary physiological function is to protect the endothelium from mechanical shear stress, leukocyte adhesion, platelet aggregation, oxidative stress and pathogen invasion [9]. Currently, little is known about its regeneration, how to prevent its damage, or the mechanisms underlying the damage. Nowadays, damage to the eGC is being recognized as part of endothelial dysfunction, which occurs in many organs, including the coronary, renal, and retinal circulation. Endothelial dysfunction underlies the development and maintenance of hypertension, which is a major cause of chronic kidney disease (CKD). It is characterized by increased oxidative stress and endothelial activation, intertwined with the immune cell response. This state of low-grade inflammation may cause damage to the eGC. However, understanding of these processes and their effects on the eGC is only just emerging. In this review, we focus on the eGC in diseases such as CKD and hypertension, and how oxidative stress and inflammation affect the eGC. A comprehensive literature search was conducted using the PubMed database to identify relevant studies. The search strategy employed specific keywords and MeSH terms, including: “endothelium; glycocalyx; hypertension; oxidative stress; renal insufficiency; chronic”. Inclusion criteria were limited to peer-reviewed original research and review articles published in English, with no restriction on date of publication whatsoever.

### 1.1. Structure, Composition and Basic Functions of eGC

**Figure 1:**
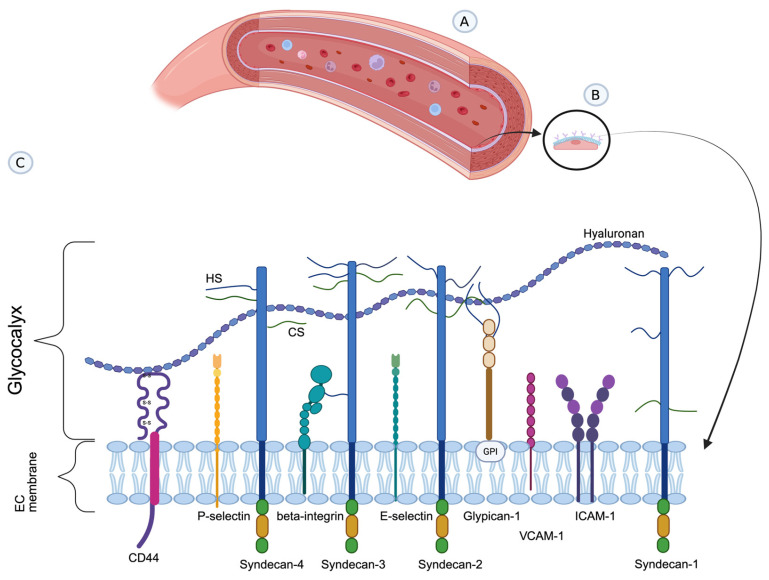
(**A**) A blood vessel model showing circulating blood cells. (**B**) The endothelial glycocalyx (eGC) covers the apical surface of the endothelial cell (EC). (**C**) Structure of the eGC. Simplified schematic of the major glycocalyx components covering the apical surface of the EC. (1) Hyaluronan (HA) (blue-purple) free proteoglycans (PGs) bind to the CD44 membrane receptor; (2) Four examples of transmembrane PG syndecans carry long glycosaminoglycan (GAG) side chains—heparan sulfate (blue, HS) and chondroitin sulfate (green, CS). In syndecan-1 and syndecan-3, both HS and CS chains can be represented, but only HS is usually represented in syndecan-2 and syndecan-4; (3) The membrane PG Glypican-1 (brown) is attached to the surface via a glycosylphosphatidylinositol (GPI) anchor and binds only one GAG-HS (blue); (4) Other glycoproteins (P-selectin, E-selectin, integrin, VCAM-1, and ICAM-1) have shorter, unbranched carbohydrate side chains. Created with BioRender.com.

The eGC is a negatively charged, carbohydrate-rich gel consisting of various glycoproteins (GPs), proteoglycans (PGs), and glycolipids (Figure 1) [7]. Visualizing the eGC under a microscope is often challenging. Assessing its thickness can be very difficult due to its variability, especially in vitro, and it almost always depends on the model and method used. The best option is to observe live, untreated cells ex vivo or in vivo [10,11,12].

Each of the membrane components of eGC has a distinct role in vascular homeostasis. GPs are composed of covalently bound, branched oligosaccharide chains that carry a negative charge due to terminal sialic acid residues. Most proteins on the endothelial surface are membrane-bound adhesion molecules, such as integrins, selectins, and members of the immunoglobulin superfamily, which are involved in endothelial cell adhesion and signaling [4,13]. In this process, the first step in leukocyte–endothelial cell interaction during inflammation is called “leukocyte rolling.” This involves the interaction of leukocyte ligands, such as PSGL-1 mucin-like glycoprotein (P-selectin glycoprotein ligand-1), with P-selectin and E-selectin located on the eGC. P-selectin is produced and stored in the Weibel–Palade bodies of endothelial cells and is rapidly released after stimulation by agents such as thrombin and histamine. In contrast, the expression of E-selectin (CD62E) is inducible and requires de novo mRNA and protein synthesis triggered by pro-inflammatory cytokines (TNF-alpha, IL-1). These selectin-mediated rolling interactions are prerequisites for the subsequent firm adhesion (arrest) of leukocytes, which is mediated by the interaction of integrins (beta-1 and beta-2 subgroups) on leukocytes with the immunoglobulin superfamily on ECs (e.g., ICAM-1, VCAM-1) [14]. The immunoglobulin superfamily, which includes intracellular adhesion molecules, intercellular adhesion molecule 1 (ICAM-1), vascular cell adhesion molecule 1 (VCAM-1), and platelet/endothelial cell adhesion molecule 1 (PECAM-1), serves as ligands for integrins on leukocytes and platelets and is a part of the eGC [7]. Furthermore, integrins on ECs mediate platelet–endothelial cell interactions, specifically those in the beta-3 subgroup [7,13,15,16].

In contrast, PGs consist of a core protein attached to negatively charged glycosaminoglycan (GAG) side chains. GAGs are linear polysaccharide molecules composed of repeating disaccharide subunits: hyaluronan (HA), chondroitin sulfate (CS), keratan sulfate (KS), dermatan sulfate (DS), and the most abundant, heparan sulfate (HS), which accounts for 50% to 90% of GAG chains. HA is also the longest free polysaccharide not attached to a core protein and binds to CD44 molecules, which serve as membrane receptors. The core proteins are anchored to the membranes of ECs and include glypican and syndecan, while others, such as biglycan, decorin, and perlecan, are not anchored to the membrane [4,7]. Syndecans are transmembrane proteins consisting of extracellular, transmembrane, and cytosolic domains. The extracellular domain binds GAGs, mostly HS and CS [7,13,17]. Syndecans, especially syndecan-1 (SDC-1), affect the integrity of the eGC and regulate leukocyte adhesion [12]. In contrast, the second most common core protein, glypicans, are attached to the surface via a glycosylphosphatidylinositol (GPI) anchor and bind only one type of GAG, HS. GPI tends to localize HS at membrane areas, lipid rafts, and caveolae. Caveolae are membrane structures that serve as communication sites where glypican participates in signaling cascades with other molecules, such as endothelial nitric oxide synthase (eNOS) [18,19]. By producing nitric oxide (NO), eNOS has a role in mediating vasodilation in response to different hemodynamic and metabolic forces (such as an increase in flow and metabolic demand of the tissue), thus rendering the eGC as a potentially important prime contact to initiate processes leading to vasodilation (discussed further in the text).

### 1.2. Physiological Role of eGC in Microcirculation

eGC plays an important role in maintaining vascular health by regulating vascular permeability, mechanotransduction, coagulation, and leukocyte–endothelial interactions [20]. These mechanisms work together to maintain vascular homeostasis, which depends on a proper balance between glycocalyx degradation and synthesis, and inhibit inflammation, the main cause of the development and progression of cardiovascular pathology [21]. Under physiological conditions, the eGC is a dynamic and inhomogeneous structure due to various electrostatic and molecular interactions between its constituents [22,23]. A dense network of GAGs, owing to their sulfate groups, imparts a distinct negative surface charge to the luminal surface of the vascular endothelium, creating an electrostatic space that repels and weakens interactions between blood cells and the vessel wall [7,18,24,25]. In addition to the electrostatic interactions, a structural barrier formed by a dense layer of PGs is essential for preserving homeostasis. Due to their height, PGs hide the binding sites on adhesion molecules, primarily selectins and integrins, under physiological conditions. This spatial isolation mechanically modulates the adhesion of inflammatory cells and platelets to the endothelial surface, which, during inflammatory processes, when glycocalyx shedding occurs, disappears and binding sites become available (discussed further in the text) [13,22].

eGC also acts as a gatekeeper [26] and prevents intravascular leakage by blocking the passage of molecules. To explain endothelial permeability, Danielli [4] hypothesized that a thin layer, later named the eGC, covers the luminal surface of endothelial cells, but provided no optical evidence. The Starling principle must be modified in most tissues due to the eGC, except in the renal cortex, intestinal mucosa, and lymph nodes. In these tissues, fluid absorption into the capillaries is maintained because the interstitium continues to be flushed. This flushing prevents accumulation around the capillaries, which would otherwise increase oncotic pressure and inhibit reabsorption. The eGC creates a sub-glycocalyx space between the ECs and itself that is almost protein-free. This generates a local oncotic pressure between the plasma and the sub-glycocalyx space, which is lower than the interstitial oncotic pressure and leads to an inability to reabsorb fluid into continuous capillaries. Thus, tissue fluid balance is maintained by lymphatic vessels. Local oncotic pressure also helps prevent edema formation because it controls intravascular filtration [27,28,29,30]. Interestingly, two studies [31,32] have shown that shedding of SDC-1 and HS does not affect vascular permeability. In contrast, degradation of HA affects endothelial dysfunction and increases vascular permeability [31].

Mechanotransduction in the microcirculation is a key process in which vascular endothelial cells, after receiving mechanical stimuli such as shear stress, convert these signals into biochemical signals that regulate vascular homeostasis [33]. As the eGC is the first structure affected when blood passes through a vessel, maintaining its integrity is important. Mechanical stimuli (shear stress) initially affect HS on glypican-1. This signal is transferred to the intracellular tail of PECAM-1, activating it. That activation triggers a kinase to phosphorylate the eNOS enzyme, an isoform of NOS mostly expressed in ECs, at the Ser1177 residue. eNOS increases NO production from L-arginine, leading to the relaxation of vascular smooth muscle cells by activating cyclic guanosine monophosphate (cGMP), thereby causing vasodilation of the vessel wall. Regulation of vascular tone depends on the secretion of NO, a vasodilator, and endothelin-1, a vasoconstrictor [33,34,35]. eNOS cannot be upregulated after removal of HS by heparinase III treatment [36], and removal of other glycocalyx components also impairs flow-mediated vasodilation [37,38,39]. This supports the role of eGC in the maintenance of vascular reactivity to stimuli.

## 2. Role of eGC in Pathophysiological Conditions

**Figure 2:**
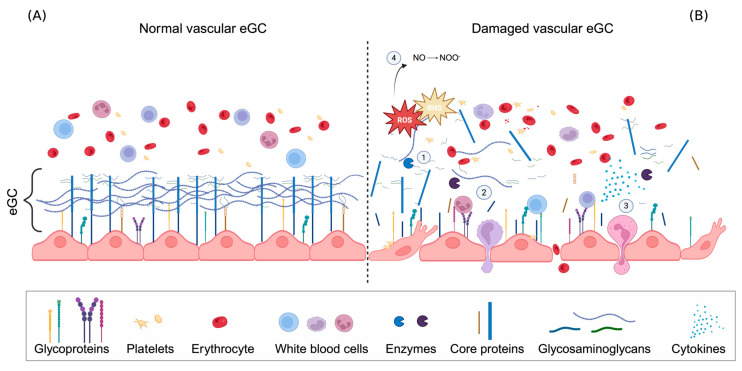
Appearance of the vascular eGC under physiological conditions (**A**) and in conditions where significant shedding of its components has occurred (**B**). Normal vascular eGC consists of core EG proteins, bound or adhered glycosaminoglycans (GAGs), and glycoproteins (GPs). Proteoglycans (PGs), composed of a core protein and GAGs, due to their height, disrupt leukocyte adhesion under physiological conditions by hiding binding sites on selectins and integrins. When oxidative stress occurs, commonly observed in conditions such as hypertension, chronic kidney disease (CKD), and diabetes mellitus (DM), which trigger a cascade of modifications and enzymatic shedding that structurally degrade the endothelial glycocalyx (eGC), exposing adhesion molecules (1) and initiating inflammatory responses. This process is followed by leukocyte rolling, adhesion, (2) and diapedesis, which are further promoted by cytokines (3). Additionally, oxidative stress impairs nitric oxide (NO) signaling, resulting in vasoconstriction (4). Created with BioRender.com.

The eGC is highly susceptible and sensitive to various pathological stimuli, such as infection, hypoxia, hypovolemia, hypervolemia, ischemia, and excessive glycemia [20,40]. Its degradation, known as “GC shedding”, is an early and central event in the development of inflammation, endothelial dysfunction, and other inflammation-related diseases (Figure 2) [40,41,42]. Loss of glycocalyx integrity leads to increased endothelial permeability, inflammation, oxidative stress, and impaired microcirculatory function, all of which strongly correlate with disease progression [41,43]. The eGC is easily disrupted by changes in the microenvironment, especially the accumulation of reactive oxygen species (ROS). The presence of ROS initiates a cascade of modifications and enzymatic shedding that structurally degrade the eGC, impair its function, and trigger or enhance the inflammatory response [41,44].

### 2.1. Oxidative Stress—The Chemical Trigger

Vascular oxidative stress is a major contributor to eGC degradation and endothelial dysfunction [45]. However, oxidative stress is not an isolated phenomenon; rather, it represents a broader manifestation of cellular and tissue distress, encompassing mitochondrial dysfunction, endoplasmic reticulum stress, DNA damage, and autophagic or lysosomal signaling pathways [46]. Also, elevated ROS levels originate from various endogenous and exogenous sources and act as triggers and mediators for inflammation by overwhelming antioxidative defenses and activating several signaling pathways (e.g., the cyclooxygenase (COX) pathway) [47,48]. During ROS influx, nuclear factor kappa B (NF-κB) is released from its inhibitor IκB and is transferred to the cell nucleus in its active form [48]. Together with transcription factors such as HIF-1α, Nrf2, AP-1, and PPAR-γ, NF-κB induces genes encoding inflammatory proteins, CAMs, growth factors, cytokines, and chemokines [49,50,51]. Cytokines and chemokines, the main drivers of the inflammatory response, subsequently induce the recruitment of polymorphonuclear neutrophils (PMNs), macrophages, and dendritic cells [52]. Additionally, oxidative stress impairs NO signaling through the production of peroxynitrite (ONOO-) [53,54].

ROS degrade GAGs and, subsequently, the PG structure of the eGC itself, contributing to impaired synthesis of its components [17,55]. The eGC is highly vulnerable to oxidative stress due to its abundance of negatively charged GAGs, which are prone to ROS-induced fragmentation, compromising structural integrity and redox homeostasis [55,56,57]. ROS and reactive nitrogen species (RNS) directly degrade the structure of non-sulfated, free GAG HA [58,59,60] and the membrane-anchored core protein SDC-1 [61,62]. Superoxide anions (O_2_^•−^) and hydroxyl radicals (•OH) drive GAG degradation (e.g., HA, HS). In the presence of iron, superoxide generates highly reactive hydroxyl radicals that catalyze molecular fragmentation [59,63,64,65].

Furthermore, the presence of ROS induces the activation of the enzyme heparanase-1 (HPR-1), which degrades HS [44,66,67]. Cleavage of HS indirectly triggers SDC-1 shedding by upregulating matrix metalloproteinases (MMPs), which degrade matrix proteins during tissue remodeling [68,69]. Increased levels of gelatinase A (MMP-2) and gelatinase B (MMP-9), the main agents in SDC-1 shedding, have been linked to the development of diabetes and other metabolic disorders [68,70,71,72]. A Disintegrin and Metalloproteinases (ADAMs), zinc-dependent endopeptidases also known as ‘sheddases’, also contribute to eGC degradation and are activated by ROS [73]. While MMPs degrade the extracellular matrix, ADAMs act as molecular scissors to physically cleave membrane-bound proteins from the cell surface, such as cytokines, growth factors, adhesion molecules and receptors [74,75]. ADAM-10 is responsible for the shedding of cell-surface proteins, while ADAM-17, also known as tumor necrosis factor-α-converting enzyme (TACE), is primarily induced by inflammation (TNF-α) and sheds inflammatory cytokines and growth factors [76]. While ROS mainly activate the enzyme cascade, proteases are the main mediators of structural damage to the eGC layer and the loss of antioxidant defense, which weakens the barrier and disrupts vascular homeostasis [41].

Through direct and indirect damage to the eGC, oxidative stress induces endothelial dysfunction and triggers inflammatory cascades. This creates a vicious cycle that amplifies vascular impairment and disease progression [41,44].

### 2.2. Inflammation—The Cellular Response

Leukocyte rolling, as we mentioned before in the text, is an initial step in the immune response, characterized by the ‘rolling’ of white blood cells (WBCs) along the inner layer of the endothelium towards sites of inflammation, before they migrate into the surrounding tissue [14,77,78]. Selectins (E-selectin, L-selectin, and P-selectin) mediate this rolling, while adhesion and subsequent migration are facilitated by interactions between leukocyte integrins and endothelial adhesion molecules (VCAM-1, ICAM-1, and PECAM-1) [14,79]. Structural impairment of the eGC leads to the loss of its barrier and adhesion regulatory functions. Adhesion molecules, initially masked, become exposed as the eGC thins, resulting in increased leukocyte–endothelium interactions [80,81]. This further compromises the eGC structure mechanically and enzymatically through protease activation and increased ROS and RNS production [41,82]. Leukocyte–endothelial interactions become exaggerated, increasing immune cell recruitment and contributing to inflammatory and autoimmune disease pathogenesis [81].

Pro-inflammatory cytokines such as TNF-α, IL-1β, and IL-6 further upregulate the activity and production of MMP, ADAM, and HPR enzymes [55,71,83,84,85]. Fragments of the damaged eGC (SDC-1 or HA) act as damage-associated molecular patterns (DAMPs), promoting endothelial dysfunction and perpetuating the cycle of oxidative stress, inflammation, and eGC shedding [71,86,87]. DAMPs exacerbate vascular injury by binding to toll-like receptors (e.g., TLR4), triggering secondary activation of the NF-κB pathway [88,89]. This closes the loop: ROS-induced eGC shedding generates DAMPs, which further activate cytokines and enzymes involved in inflammation [89,90].

Cytokines further promote selectin and integrin expression, encouraging leukocyte rolling, adhesion, and diapedesis [91]. Diapedesis is the final step of the immune response, during which leukocytes (neutrophils) migrate through the endothelium via transcellular route or paracellular routes into the surrounding tissue [92,93]. These processes cause eGC thinning and degradation, increasing transmigration and promoting a pro-migratory phenotype [94]. This endothelial phenotype, characteristic of chronic low-grade systematic inflammation [46], leads to a permanent reduction in NO production and bioavailability, which further activates the NF-κB pathway [79,94]. The shift from acute eGC shedding to chronic inflammation marks a transition in vascular pathology [95]. The eGC plays a central role in this process, acting as both a target and mediator of the immune response and driving vascular remodeling and disease progression [39]. In the pathogenesis of CKD, canonical inflammation, such as massive migration of leukocytes into the tissue, and chronic low-grade inflammation can develop, creating a vicious pathogenic cycle [46]. In conditions such as CKD and hypertension, this cycle of oxidative stress, inflammation and eGC degradation is continuously reinforced, leading to long-term vascular dysfunction.

### 2.3. Effect of Metabolic and Environmental Factors

eGC degradation is simultaneously driven by metabolic (intrinsic) and environmental (extrinsic) factors, resulting in a characteristic pathophysiological pattern of increased permeability, a pro-inflammatory and pro-coagulant state, microcirculatory dysfunction, and consequent organ damage [96]. Metabolic influences on the eGC include chronic disturbances of glucose, lipid profile, blood pressure, body weight, renal function, and systemic inflammation, which alter the chemical and mechanical environment of the endothelium and promote thinning, fragmentation, and shedding of the eGC [18]. For example, in chronic hyperglycemia and insulin resistance, excess glucose leads to the formation of advanced glycation end products (AGEs) and activation of their receptors (RAGE) on endothelial cells, enhancing the generation of ROS and triggering inflammatory signaling pathways. Similarly, dyslipidemia and an atherogenic profile (elevated LDL/oxLDL, hypertriglyceridemia) promote oxidative stress and NF-κB activation, driving enzymatic degradation of the eGC [24,41,97]. In metabolic syndrome and obesity, excessive adipose tissue releases pro-inflammatory adipokines (TNF-α, IL-6), sustaining chronic low-grade inflammation and oxidative stress, which further enhances protease activity and impairs synthesis of new glycocalyx components [18,79,98]. In patients with diabetes, AGEs can accumulate in long-lived proteins such as vascular collagen and alter arterial elasticity [99,100]. One study [101] found that patients with type 2 diabetes have less distensible arteries than normal, and those with both diabetes and hypertension have greater arterial stiffness. Additionally, endothelial dysfunction and reduced bioavailability of NO occur, leading to impaired vascular tone [99]. Research has shown that diabetes and hypertension interact synergistically, accelerating the progression to kidney dysfunction in rat models [102] and increasing the risk of cardiovascular disease (CVD) due to endothelial dysfunction [21]. Ultimately, strict control of blood pressure and blood glucose levels remains fundamental in preventing the development of severe kidney damage, as diabetes and hypertension are leading causes of CKD [102]. However, this synergy has not been consistently observed in human populations. For example, a 10-year cohort study [103] found no significant multiplicative or additive interaction between these two conditions regarding the incidence of CKD. This translational gap highlights the risks of extrapolating results from animal models to humans, where genetic and environmental factors may play a more dominant role. Further prospective human research is needed to clarify these complex interactions and refine clinical prevention strategies.

Environmental factors are external influences arising from lifestyle or the surrounding environment (smoking, diet, physical activity, air pollution, hemodynamic and hypoxic stress) that, through oxidative stress, mechanical strain, and inflammation, further damage the glycocalyx or amplify the effects of metabolic disturbances. The main categories include smoking and air pollution, unhealthy diet, physical inactivity versus excessive acute loading, hemodynamic stress (e.g., abrupt changes in blood pressure and flow), and hypoxic–reperfusion stress (ischemia–reperfusion, heat stress, and severe hypoxia) [44,104]. Smoking and exposure to polluted air introduce particles and reactive chemicals into the body that generate ROS and trigger systemic inflammation. An unbalanced diet, particularly frequent postprandial spikes in glucose and lipids combined with low antioxidant intake, further sustains chronic oxidative stress and low-grade inflammation, persistently shifting the balance between synthesis and degradation of the glycocalyx towards degradation. However, the current literature provides firm evidence only from animal studies, not from humans, and raises further hypotheses for future research. Physical inactivity indirectly contributes to these mechanisms by worsening insulin sensitivity, dyslipidemia, and inflammation, whereas extreme acute loading or repeated exposure to very high hemodynamic forces (e.g., abrupt blood pressure oscillations, high turbulent flow) can mechanically damage the eGC [41,104].

These environmental determinants do not act in isolation but are superimposed on existing metabolic disturbances, accelerating the transition from a compensated state to overt endothelial dysfunction, which leads to disease development.

## 3. eGC in Hypertension and CKD

CKD is a common global health issue and one of the leading causes of death worldwide, but most deaths are due to cardiovascular complications rather than progression to end-stage renal disease (ESRD) [105]. Although the mortality rate from CVD has decreased, this improvement has not occurred in the CKD population [105,106]. The risk of CVD in CKD cannot be explained solely by traditional Framingham risk factors (age, sex, smoking, high blood pressure, cholesterol and glucose levels, family history, and obesity), but also by new factors such as oxidative stress, endothelial damage and vascular calcification [107,108]. Additionally, research indicates that endothelial damage can play an important role in primary hypertension that leads to CVD, which is one of the main causes of CKD.

Loss of eGC integrity is a primary cause of microvascular dysfunction, one of the earliest changes in the pathogenesis of CVD [109]. As mentioned previously, the endothelium plays a crucial role in regulating vascular tone through the balanced release of vasoreactive molecules, such as NO. Reduced bioavailability of NO has also been implicated in the development of hypertension [110]. Hypertension in CKD is a multifaceted condition which includes the loss of NO bioavailability driven by systemic endotheliosis and the overproduction of vasoconstrictors by activated ECs and dysfunction of the renin–angiotensin–aldosterone system (RAAS) related to renal pathology. Excessive RAAS activation, triggered by reduced renal perfusion, causes systemic vasoconstriction and sodium retention. These processes contribute to endothelial dysfunction and accelerate the development of cardiovascular and renal diseases [111].

The eGC can act as a sodium buffer because of its negative charge due to its sulfated GAGs. This allows the eGC to bind to positively charged particles, including sodium ions, and protect cells from damage caused by excessive sodium. The epithelial sodium channel (ENaC) regulates nanomechanics: when sodium influx increases, it raises the mechanical stiffness of the cellular cortex [112], which emphasizes that sodium homeostasis and salt sensitivity appear to be related not only to renal dysfunction but also to endothelial dysfunction [112,113]. Sodium overload (135 to 150 mM) damages the eGC by causing the loss of some HS residues. As a result, the eGC loses its sensitivity to blood flow and its elasticity, leading to reduced production of NO and progression to hypertension [112,114,115,116]. Refs. [117,118] observed that arterial stiffness is a predictor of hypertension. Soluble markers of eGC (HA and SDC-1) are independently associated with aortic stiffness and microcirculatory function in patients with hypertension [119]. Endothelial function in larger vessels, as evaluated by flow-mediated vasodilation (FMD), does not have any association with endothelial function in the microcirculation because of the heterogeneity of endothelial dysfunction across different vascular beds [119]. A slight increase in sodium concentration in human vascular smooth muscle cells (hVSMCs) leads to the degradation of the membrane eGC but does not affect the whole cell. Long-term exposure significantly decreases the thickness of the eGC and causes hypertrophy of the cells, which may reduce the arterial lumen and lead to hypertension [120]. In animal models, Zheng et al. observed that a high-salt diet leads to reduced microvascular properties, increased perfused boundary region (PBR) [121], and significant disruption of the eGC (dysregulation of integrins) [122].

A damaged eGC allows erythrocytes to penetrate deeper into the endothelium, as manifested by an increased PBR [109,123]. Ikonomidis et al. [124] described an association between glycocalyx damage (PBR) and markers of arterial stiffness (PWV and cSPB) in participants with untreated hypertension compared with healthy controls. They suggest that eGC thickness is reduced in untreated hypertension [124]. Vlahu et al. [125] observed that dialysis patients had significantly increased PBR and soluble HA and SDC-1 markers compared with healthy controls. The same dialysis patients were divided into two groups (with and without CVD), and those with CVD had increased PBR compared with those without CVD. However, there was no significant increase in soluble HA and SDC-1 markers of eGC in this comparison [125]. In a prospective observational study [126], it was noted that PBR is associated with the risk of future cardiovascular events in patients without established CVD [126]. In addition, PBR and SDC-1 were increased in patients with ESRD compared to controls. However, there were no differences between dialysis and non-dialysis ESRD patients. The PBR in stable transplant recipients was higher, but not statistically significant, possibly due to large variability and group heterogeneity [127]. On the other hand, according to Liew et al., they found no differences in PBR between groups (patients with CKD, dialysis patients, and kidney transplant recipients) [128]. The lack of consistency in PBR findings can be attributed to variations in eGFR stages and clinical status. While Liew et al. [128] focused on stable outpatients (median eGFR 31 mL/min/1.73 m^2^), studies reporting significant glycocalyx impairment (Dane et al. [127] and Vlahu et al. [125]) involved critically ill or transplantation-eligible patients with advanced renal failure (mean eGFR < 8.2 mL/min/1.73 m^2^). These findings imply that structural glycocalyx degradation, as measured by PBR, may only reach detectable thresholds during acute illness or advanced stages of kidney dysfunction. However, in a study by Liew et al. [128], there was a large variability in PBR readings within each group, which may account for the limited difference between them. Notably, despite a smaller sample size of Dane et al. [127] compared to Liew et al. [128], it showed significant differences in PBR, suggesting that disease severity may outweigh sample size in determining PBR sensitivity. Biological heterogeneity can also be explained by anatomical variability, as it is debatable whether one can extrapolate the changes in one microcirculatory bed to another. For instance, a porcine study did not show a correlation between the sublingual and renal cortical microcirculation on the kidney [129], but only showed a correlation when the disease became more systemic [130]. Additional sources of variability are methodological factors, such as operator dependence (focus, probe pressure), and the influence of external factors, such as caffeine intake, smoking, or fasting. A source of variability can also be the rheological properties of blood (changes in RBC deformability), because end-stage renal disease is associated with alterations in RBC mechanical properties and changes in rheology [131]. Consequently, strict methodological standardization remains essential to overcome the high variability of PBR measurements and confirm their predictive value in routine clinical practice. However, markers of eGC damage (SDC-1) and PBR were higher in dialysis patients and returned to normal after 3 months from transplantation, similar to healthy controls, but serum hyaluronan levels did not change and remained low [132]. In a meta-analysis including 2201 ESRD patients, Gansevoort et al. [133] found that lower eGFR and higher albuminuria are associated with ESRD. Altogether, these studies suggest that assessing eGC damage through the PBR may serve as a crucial biomarker for evaluating vascular risk and the progression of renal diseases.

Serum markers of the eGC (HA and SDC-1) and endothelial dysfunction worsen with CKD progression [119,128]. HA and SDC-1 are also independently associated with vascular dysfunction regardless of age. On the other hand, in an elderly community cohort, including cardiovascular risk factors, no associations were observed between eGFR and system circulation [119]. In patients with ESRD, the optimal treatment is kidney transplantation, which requires assessment of the organ’s condition; therefore, the quality of donated organs is crucial. One retrospective observational cohort study showed a positive correlation between serum SDC-1 levels and creatinine before organ procurement. SDC-1 was also higher in patients with acute kidney injury than in those without such injury. SDC-1 may be a biomarker for assessing the quality of donated organs [134]. Serum SDC-1 is longitudinally, but not directly, associated with declining kidney function and increased cardiovascular risk in patients with idiopathic nephrotic syndrome (INS), which can progress to CKD and cardiovascular morbidity [135].

The underlying mechanisms of glycocalyx damage and endothelial activation in CKD are multifactorial and include hypertension, low-grade inflammation and uremic toxicity [127,136]. Claro et al. show that circulating uremic toxins (indoxyl sulfate, IS, and p-cresyl sulfate, p-CS) increase the production of MCP-1, a molecule that promotes inflammation in blood vessels. This may explain why blood vessels in patients with CKD are more prone to inflammation [136]. Nakagawa et al. suggested that in isolated rat thoracic aortas, the uremic toxin, indoxyl sulfate, can cause accumulation of ROS, which reduces vasodilation induced by rapid response agents (ACh and SNP) through NO inactivation [137]. The use of an oral adsorbent has been shown to reduce the accumulation of uremic toxins, slow the deterioration of renal function, and improve the endothelial function in a rat CKD model [138]. Perlecan (HP PG) and decorin (leucine-rich PG) are glycocalyx components that may be linked to atherosclerotic plaque formation and vascular calcification. One cross-sectional study has shown how perlecan and decorin change in patients with CKD. Perlecan and decorin were higher in patients with stage 4–5 CKD, possibly due to endothelial damage, while decorin levels were lower in transplant recipients than in those with CKD. Plasma perlecan levels also return to the optimal range in patients who undergo kidney transplantation [139]. In an animal model, Ermet et al. observed a reduction in the thickness and density of the eGC on peritubular capillaries in murine kidneys during CKD [140].

Key findings from the above studies are presented in Table 1. This cross-section shows that eGC damage is directly associated with the progression of renal disease, with levels of “shedding” markers (SDC-1, HA) correlating with the accumulation of uremic toxins and vascular complications. Although the damage is most pronounced in patients on dialysis, successful kidney transplantation enables normalization of the glycocalyx barrier, confirming its reversibility and potential as a therapeutic target.

## 4. Perspective and Therapeutic Implication

The glycocalyx could serve as a powerful tool for monitoring vascular conditions. Early detection of vascular changes is crucial for preventing irreversible organ damage. In many critical conditions, such as trauma, sepsis, coronavirus disease, acute respiratory distress syndrome, kidney diseases, cardiovascular disorders, autoimmune diseases, cancer, pregnancy-related complications or inflammation, its components are released into the bloodstream and could be used as diagnostic and prognostic biomarkers [141]. Research involving the eGC and its fragments, such as SDC-1, HA, HS and endocan, which serve as biomarkers of damage, represents a valuable diagnostic indicator of disease progression and a potential pharmacological target [43,56,142,143,144,145,146]. Elevated levels of SDC-1 are significantly associated with the development of sepsis [141]. Regarding sepsis severity and prognostic significance, endocan demonstrated high accuracy as a mortality biomarker [147]. High levels of HS indicate poor neurological outcomes in resuscitated patients [56]. In acute respiratory distress syndrome (ARDS) and Kawasaki disease, HA values are elevated and strongly associated with coronary artery disease, the most serious complication of Kawasaki disease [148,149]. Therefore, future research should focus on expanding our understanding of the role of eGC-derived biomarkers in disease mechanisms and refining their clinical application through standardized cutoff thresholds, disease-specific reference ranges, and validation of their utility in randomized controlled trials.

The endothelial glycocalyx-degrading enzyme heparinase contributes to vascular leakage and inflammation. Buijsers et al. showed that low-molecular-weight heparins (LMWHs), which act as heparinase inhibitors, can reduce heparinase activity in patients with COVID-19 (coronavirus disease-2019) and may improve clinical outcomes [150]. In vitro experiments using synthetic membranes and human glomerular endothelial cells have further demonstrated that albumin at physiological concentrations is crucial for preserving glycocalyx integrity, as it prevents the degradation of SDC-1 and HS induced by MMP-9 and heparinase [151]. Similarly, in vitro findings from rat endothelial cells suggest that sphingosine-1-phosphate (S1P) protects the glycocalyx by inhibiting the MMP-dependent shedding of the SDC-1 ectodomain [152], but it also restores the eGC once damaged [39]. For clinical application, meta-analyses of randomized controlled trials (RCTs) have confirmed that SGLT-2 inhibitors significantly improve endothelial function in patients with type 2 diabetes by reducing oxidative stress and vascular inflammation [153]. Furthermore, in vivo animal models of sepsis have shown that recombinant thrombomodulin (rhTM) protects against acute respiratory distress syndrome by preserving the synthesis of glycocalyx components [154].

Taken together, glycocalyx components could serve as important biomarkers for the early detection of life-threatening conditions and for the assessment of treatment outcomes. Although glycocalyx degradation occurs during trauma or critical illness, it represents only one of many pro-inflammatory and pro-thrombotic processes and must be considered alongside other microvascular changes, such as endothelial dysfunction, leukocyte and platelet adhesion, and the release of inflammatory mediators such as cytokines and ROS [81].

## 5. Conclusions

Based on these observations, eGC damage, known as “glycocalyx shedding”, appears to be a primary cause of endothelial dysfunction in CKD. However, future studies should clarify whether this shedding serves as an early biomarker, a secondary consequence of hypertension and renal disease, or results from pathological changes in the microcirculation that lead to organ failure. Overall, data from microcirculation experiments in patients with CKD indicate that biochemical markers like SDC-1 are more consistent indicators of early injury, whereas PBR alterations typically reach detectable thresholds only during advanced or acute disease stages. The improvement of glycocalyx integrity following successful kidney transplantation underscores the potential of these parameters for monitoring therapeutic response and evaluating vascular vulnerability.

## Figures and Tables

**Table 1:** Cross-section of research studies of biomarkers of endothelial function, including components of eGC in chronic kidney disease.

Participants	Study Design	Targets	Effects	Limitations	References
hypertension patients with a wide range of eGFR (*n* = 107)	cross-sectional data from two randomized clinical trials	HA, SDC-1, cfPWV	Associations of HA with aortic stiffness and SDC-1 with endothelial dysfunction.	The sample size limited the number of potential confounding factors. A limited number of circulating biomarkers of vascular endothelial function and markers of eGC proteins.	[119]
patients with ESRD (*n* = 40; 17 PD and 23 HD)	cross-sectional observational study	HA, SDC-1 PBR, CRP	↑ PBR were increased in patients compared with healthy control (*p* < 0.01); ↑ CRP = ↑ PBR (*p* = 0.03); ↑ serum levels of HA, SDC-1 were higher in patients compared with healthy controls (*p* < 0.01).	Study design. ESRD is associated with alterations in RBC mechanical properties and changes in rheology.	[125]
patients with ESRD, eGFR < 15 mL/min na 1.73 m^2^ (*n* = 23); normal kidney function after successful living donor kidney transplantation, eGFR = 30 mL/min na 1.73 m^2^ (*n* = 12); patients who developed interstitial fibrosis/tubular atrophy after kidney transplantation, eGFR < 30 mL/min na 1.73 m^2^ (*n* = 10)	cross-sectional observational study	PBR, SDC-1	↑ PBR and SDC-1 were inversely correlated with the ↓ eGFR (*p* < 0.05); the perfused boundary region in transplant patients was indistinguishable from that of healthy controls; patients who developed interstitial fibrosis and tubular atrophy showed a ↑ PBR (*p* < 0.01).	The effect of immunosuppression in the transplant patients on the mechanical properties of the ESL.	[127]
dialysis group (*n* = 33, PD and HD); patients with CKD, eGFR < 60 mL/min/m^2^ (*n* = 32); kidney transplant recipients (*n* = 30)	cross-sectional observational study	SDC-1, HA, vWF, VCAM-1, IS, p-CS	Serum hyaluronan negatively correlated with eGFR (r = −0.47, *p* < 0.001); HA and SDC-1 positively correlated with VCAM-1, vWF, IS, and p-CS (*p* < 0.0001).	A small sample size. Study design.	[128]
children and adults with INS (*n* = 348)	prospective cohort study	SDC-1	Serum SDC-1 positively correlated with proteinuria, but not with eGFR.	The lack of serial measurements of serum SDC-1. The limited follow-up time.	[135]

eGFR—mean estimated glomerular filtration rate; HA—hyaluronan; SDC-1—syndecan 1; cfPWV—carotid–femoral pulse wave velocity; eGC—endothelial glycocalyx; ESRD—end-stage renal disease; PD—peritoneal dialysis; HD—hemodialysis; PBR—perfused boundary region; CRP—C-reactive protein; RBC—red blood cells; ESL—endothelial surface layer; CKD—chronic kidney disease; vWF—von Willebrand factor; VCAM-1—vascular cell adhesion molecule; IS—indoxyl sulfate; p-CS—p-cresyl sulfate; INS—idiopathic nephrotic syndrome. ↑ means increase; ↓ means decrease.

## Data Availability

No new data were created or analyzed in this study. Data sharing is not applicable to this article.

## Abbreviations

ADAMs	A Disintegrin and Metalloproteinases
AGE	advanced glycation end products
ARDS	acute respiratory distress syndrome
cGMP	cyclic guanosine monophosphate
CKD	chronic kidney disease
COVID-19	coronavirus disease-2019
CS	chondroitin sulfate
CVD	cardiovascular disease
DAMPs	damage-associated molecular patterns
DS	dermatan sulfate
ER	endoplasmic reticulum (ER)
ECs	endothelial cells
eGC	endothelial glycocalyx
eNOS	endothelial nitric oxide synthase
ENaC	epithelial sodium channel
ESRD	end-stage renal disease
FMD	flow-mediated vasodilation
GAG	glycosaminoglycan
GPI	glycosylphosphatidylinositol
GPs	glycoproteins
HA	hyaluronan
HPR-1	heparanase-1
HS	heparan sulfate
hVSMCs	human vascular smooth muscle cells
ICAM-1	intercellular adhesion molecule 1
INS	idiopathic nephrotic syndrome
IS	indoxyl sulfate
KS	keratan sulfate
LMWHs	low-molecular-weight heparins
MMPs	matrix metalloproteinases
NF-κB	transcription factor nuclear factor kappa B
NO	nitric oxide
p-CS	p-cresyl sulfate
PBR	perfused boundary region
PECAM-1	platelet/endothelial cell adhesion molecule 1
PGs	proteoglycans
PMNs	polymorphonuclear neutrophils
PSGL-1	P-selectin glycoprotein ligand-1
RAAS	renin–angiotensin–aldosterone system
rhTM	recombinant thrombomodulin
RNS	reactive nitrogen species
ROS	reactive oxygen species
SDC-1	syndecan-1
S1P	sphingosine-1-phosphate
TACE	tumor necrosis factor-α-converting enzyme
TLRs	toll-like receptors
VCAM-1	vascular cell adhesion molecule 1
WBCs	white blood cells
